# The anti-aging effects of Renshen Guben on thyrotoxicosis mice: Improving immunosenescence, hypoproteinemia, lipotoxicity, and intestinal flora

**DOI:** 10.3389/fimmu.2022.983501

**Published:** 2022-10-25

**Authors:** Qin Feng, Guangyan Li, Wenkai Xia, Guoxin Dai, Jidong Zhou, Yan Xu, Deshan Liu, Guimin Zhang

**Affiliations:** ^1^ Center for Pharmacological Research, State Key Laboratory of Generic Manufacture Technology of Chinese Traditional Medicine, Lunan Pharmaceutical Group Co., Ltd., Linyi, China; ^2^ Department of Traditional Chinese Medicine, Qilu Hospital of Shandong University, Jinan, China

**Keywords:** anti-aging, immunosenescence, hypoalbuminemia, lipotoxicity, intestinal flora, multiomics

## Abstract

With the rapid aging of the population, the control of age-related disease susceptibility and prognosis faces greater challenges. There is an urgent need for a strategy to maintain the vitality of elderly people. In this study, the effect of Renshen Guben (RSGB) oral liquid was investigated on an accelerated aging mice model of thyrotoxicosis by conventional detection methods combined with multiomics technology. The results showed that RSGB increased the number of neutrophils and lymphocytes, enhanced the function of lymphocytes, and increased the levels of complement and antimicrobial peptides, which indicated that RSGB improved the immunity of thyrotoxicosis mice at the cellular and molecular levels. RSGB corrected malnutrition in thyrotoxicosis mice by improving anemia, hypoalbuminemia, ion transporters, and vitamin-binding proteins. RSGB significantly reduced the lipotoxicity by reducing the level of fatty acids, triglyceride, sphingolipids, and glucocorticoids, thus increasing the level of docosapentaenoic acid (DPA) and bile acids, which contributed to improve immunosenescence. The intestinal defense ability of thyrotoxicosis mice was enhanced with the increase of bile acids and lactic acid bacteria by the RSGB treatment. The plant metabolomics analysis showed that there were various active components in RSGB oral liquid and medicated serum, including terpenoids, phenolic acids, flavonoids, tannin, alkaloids, organic acids, phenolamines, amino acids, and others. They have antioxidant, immune regulation, and anti-aging effects, which was the material basis of RSGB. Totally, RSGB protected the thyrotoxicosis mice against aging by improving immunosenescence, hypoproteinemia, lipotoxicity, and the intestinal flora. It will be beneficial for improving the disease susceptibility and prognosis of the elderly.

## 1 Introduction

The global population over the age of 65 is growing at an unprecedented rate and is expected to reach 1.6 billion by 2050. The core of the aging problem is the increase of disease susceptibility and poor prognosis, and complex drug treatment increases this risk. Mastering how to arouse and control the resilience mechanism of aging individuals may result in a new chapter to be written for human medicine (1). The COVID-19 is currently emerging as a typical elderly-related disease; the declined defense ability plays an important role in this process (2, 3). Moreover, with the increase of the frequency and time of antibiotic use, the drug resistance of microorganisms is a major problem faced by individuals and society (4). Therefore, restoring the self-resilience ability is a scientific strategy to protect elderly people from infectious diseases. Drugs or nutrients that can enhance the resilience are particularly beneficial to the health of aging people, especially in the current COVID-19 epidemic (5, 6).

There are various cells, proteins, and metabolites in the circulatory system, which are of great significance to maintain the normal physiological function of the body. In addition to immune cells, a large number of immune molecules are the first line of defense of the body, such as antibodies, complements, and antimicrobial peptides (AMPs). With the decline in immune cells, immune molecules reflect a decline in the reserve function of the body, which is responsible for the decline in resistance to diseases in the elderly (7). There are also many functional proteins, such as transporters and binding proteins. Hypoproteinemia will lead to impaired transport and clearance of metabolic waste, and toxicity from the buildup of metabolic waste products will contribute to aging—for example, lipotoxicity is one of the most striking consequences of the disruption of metabolic homeostasis (8). Lipotoxicity leads to mitochondrial dysfunction by increasing oxidative damage, which is harmful not only for the organs but also for the immune cells (9). The intestinal tract is the key target organ to maintain animal and human health. The intestinal microbiota plays a very important role in immune system activities and the production of secondary bile acids (10). An imbalance of the intestinal microbiota can lead to many age-related degenerative diseases and unhealthy aging (11). Bile acids act as signaling molecules in nutrient absorption and activate multiple receptors that help maintain tolerance in liver and intestinal immunity (12). Therefore, bile acids interact with the intestinal flora and jointly affect intestinal health. In our previous study, the levels of bile acids were found to be significantly decreased in thyrotoxicosis mice. Because of the lack of bile acids to control the intestinal flora, the intestinal defense ability may be declined in thyrotoxicosis mice. It suggests that any strategy that can regulate bile acid homeostasis and the intestinal flora balance may have great potential for maintaining immunity and anti-aging.

Renshen Guben (RSGB) is a famous classic prescription with the function of consolidating constitution and restoring vitality. The RSGB prescription is made of *Panax ginseng* (Ren Shen), *Rehmannia glutinosa* (Di Huang, dried), *Rehmannia glutinosa* (Di Huang, prepared), *Cornus officinalis* (Shan Zhu Yu), Rhizoma Dioscoreae (Shan Yao), Rhizoma Alismatis (Ze Xie), Cortex Moutan (Mu Dan Pi), *Poria cocos* (Fu Ling), *Ophiopogon japonicus* (Mai Dong), and *Asparagus cochinchinensis* (Tian Dong). Due to its long-term traditional use and well-known efficacy, these herbal medicines have been listed in in the Chinese Pharmacopoeia (13), and their pharmacological effects, especially the anti-aging effect, have been widely studied. The anti-aging effect is mainly attributed to their antioxidant and immunomodulatory abilities (14–23). In addition, these traditional Chinese medicines (TCMs) also contain a variety of nutritional elements, which have a direct nutritional supplement effect. It is speculated that their combined prescription RSGB will have a super anti-aging effect. Although RSGB has a wide range of clinical applications according to the TCM theory, the mechanism in modern medical theory and the active ingredient are still unclear. Appropriate animal models and indicators in line with the dialectics of TCM are very important to study the pharmacological effects of TCM prescriptions. Our previous study found that the thyrotoxicosis mice model has the characteristics of immunosenescence, malnutrition, and lipotoxicity, which can be identified as an accelerated aging model for the screening of anti-aging drugs (24). Here the anti-aging effect of RSGB oral liquid on thyrotoxicosis mice was investigated in the aspects of immunity, nutrition, and metabolism. Bile acids affect the intestinal flora, and our previous study found a significant decrease in bile acid levels in thyrotoxicosis mice, so this study continued to examine the changes in the gut flora of thyrotoxicosis mice. Some ingredients in the RSGB prescription have been reported to have the potential to modulate the intestinal microbiota (25). Therefore, the influence of RSGB on the intestinal flora was also detected. In the present study, blood routine, biochemistry, flow cytometry, proteomics, metabolomics, microbiomics, and plant metabolomics were combined to clarify the action mechanism and material basis of RSGB.

## 2 Materials and methods

### 2.1 Preparation of RSGB oral liquid

The RSGB oral liquid was provided by Lunan Hope Pharmaceutical Co., Ltd. (China). It was prepared from 10 herbs: *Panax ginseng* (Ren Shen, 23 g), *Rehmannia glutinosa* (Di Huang, dried, 46 g), *Rehmannia glutinosa* (Di Huang, prepared, 46 g), *Cornus officinalis* (Shan Zhu Yu, 46 g), *Rhizoma Dioscoreae* (Shan Yao, 92 g), *Rhizoma Alismatis* (Ze Xie, 46 g), *Cortex Moutan* (Mu Dan Pi, 46 g), *Poria cocos* (Fu Ling, 46 g), *Ophiopogon japonicus* (Mai Dong, 46 g), and *Asparagus cochinchinensis* (Tian Dong, 46 g). The preparation process was as follows: The volatile components from *Cortex Moutan* were extracted by distillation, and the drug residue and liquor were retained for further use. *Panax ginseng* and *Cornus officinalis* were prepared by refluxing 60% ethanol twice; the extracts were combined and filtered, and the filtrate and the drug residue were retained for further use. The above-mentioned two kinds of drug residues and the remaining seven herbs were decocted with water which was added for two times; the decoction was filtered, combined, and concentrated. Ethanol was added to 60% and then combined with the previous *Panax ginseng* and *Cornus officinalis* ethanol extraction solution and filtered. Ethanol was removed by concentration; then, water was added to boil and filtered. The volatile components of *Cortex Moutan* and 100 g of syrup were added, adjusted to a pH value of 7.0, and then water was added to 1,000 ml. The oral solution was obtained after sterilizing. The amount of original drug in each milliliter of oral liquid was 0. 483 g.

### 2.2 Animals and reagents

Four- to 6-week-old healthy female KM mice (SPF grade) with body weight of 16–18 g were purchased from Ji’nan Pengyue Laboratory Animal Breeding Co., Ltd. (Shandong, China). The mice were housed in a clean room at a temperature of 23 ± 2°C and humidity of 50 ± 5% with a 12-h alternating light and dark cycle. They were permitted free access to food and water. All animal experiments were performed according to the National Institutes of Health Guidelines for the Care and Use of Laboratory Animals and were approved by the Animal Care and Use Committee of Shandong Province, China (approval number: AN-IACUC-2020-047). The thyroxine tablets were purchased from Shandong Renhe Pharmaceutical Co., Ltd. (China). PerCP-Cy™5.5 Hamster Anti-Mouse CD3e (catalog number 551163), FITC Rat Anti-Mouse CD4 (catalog number 553047), APC-H7 Rat anti-Mouse CD8a (catalog number 560182), PE Rat Anti-Mouse IFN-γ (catalog number 554412), and Leukocyte Activation Cocktail, with BD GolgiPlug (catalog number 550583), were purchased from BD Pharmingen™. All chemicals and solvents were of analytical or HPLC grade. Water, methanol, acetonitrile, formic acid, pyridine, n-hexane, methoxylamine hydrochloride, and N,O-bis (trimethylsilyl) trifluoroacetamide with 1% chlorosilane were purchased from CNW Technologies GmbH (Germany), while L-2 chlorophenylalanine was from Shanghai Hengchuang Biotechnology Co., Ltd. (China).

### 2.3 Animal experimental design

#### 2.3.1 Dose-dependent study

The mice were randomly divided into five groups: control group (c), vehicle treatment model group (v), and three-dose RSGB treatment group (l, m, and h, respectively). For l, m, and h RSGB treatment groups, the RSGB oral liquid was administrated at intragastric doses of 5, 10, and 20 ml·kg^-1^ once a day. The model group was administered with vehicle (purified water). Then, 10 days later, at half an hour after RSGB administration, thyroxine tablet suspension was given by an intragastric dose of 320 mg·kg^-1^, once a day, for 10 days. At the end of the experiment, the animals were subjected to fasting on the night before dissection. Blood was taken from the main abdominal vein after the administration of anesthesia with 60 mg·kg^-1^ pentobarbital sodium. Blood was injected to EDTA-K2 vacuum tubes for routine blood test.

#### 2.3.2 Mechanism research

**Figure 1:**
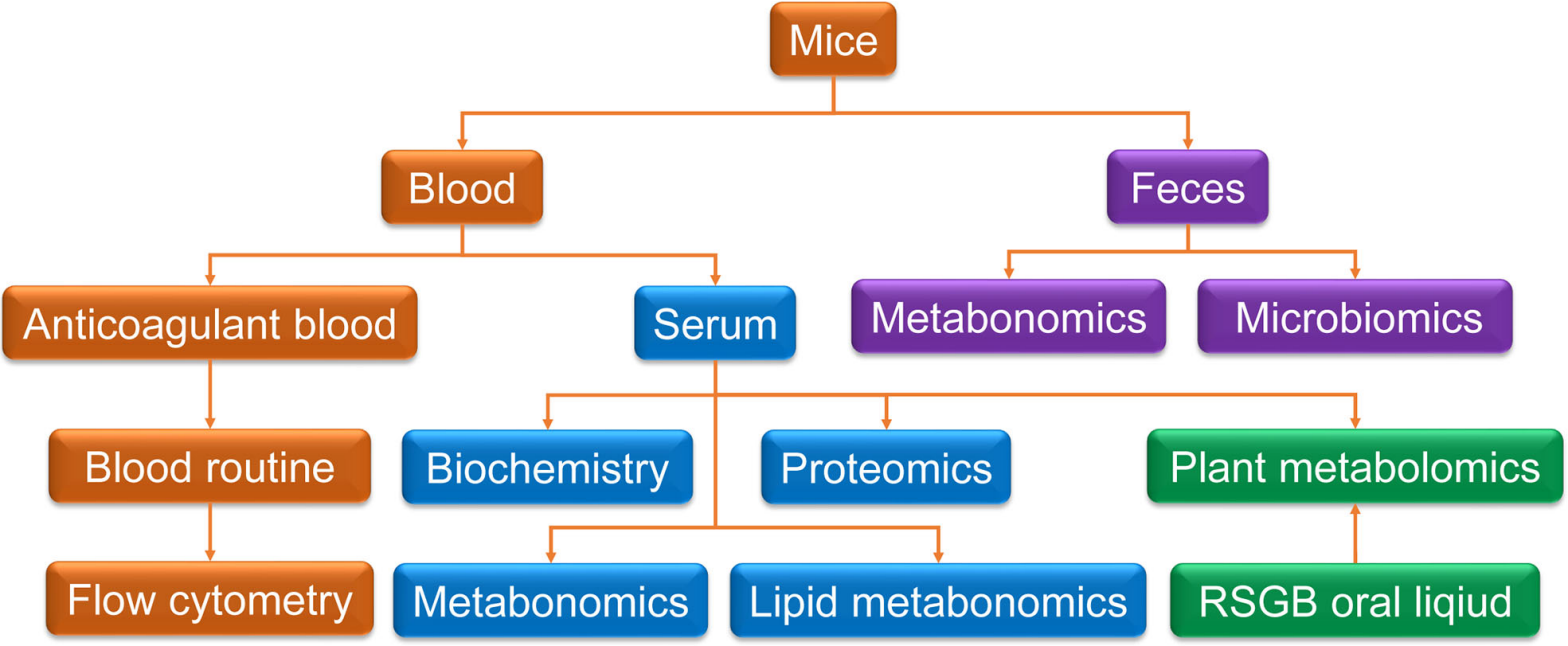
Scheme of the methodology. Blood and feces were collected for multi-technology detection. Anticoagulant blood was used for routine blood test and flow cytometry. Serum was taken for biochemistry detection and proteomics and metabolomics detection. Feces were collected for metabolomics and microbiomics. Plant metabolomics was used for Renshen Guben oral liquid and drug-containing serum.

The mice were randomly divided into three groups: control group (C), vehicle treatment model group (V), and RSGB treatment model group (L). For L group, the RSGB oral liquid was administrated by an intragastric dose of 5 ml·kg^-1^ twice a day. The model group was administered with vehicle (purified water). At 10 days later, half an hour after RSGB administration, thyroxine tablet suspension was given by an intragastric dose of 320 mg·kg^-1^ at once a day for 20 days. Feces were collected before the end of the experiment and cryopreserved at -80°C for metabolomic examination. At the end of the experiment, the animals were subjected to fasting on the night before dissection, and blood was taken from the main abdominal vein after the administration of anesthesia with 60 mg·kg^-1^ pentobarbital sodium. About 300 ul blood was injected to EDTA-K2 vacuum tubes for routine blood test and flow cytometry. The remaining blood was injected to a gel separation tube, and the serum was separated after centrifugation at 2,000 *g*. Serum was taken out for biochemical detection and proteomics and metabonomics detection. The scheme of the methodology is shown in **Figure 1**.

In order to investigate the safety of the RSGB oral liquid, the blood routine, biochemical, and pathological indexes of mice were detected in mice treated with RSGB oral liquid alone. The mice were randomly divided into two groups: control group (N) and RSGB group (W). For the W group, the RSGB oral liquid was administrated by an intragastric dose of 20 ml·kg^-1^ at twice a day for 30 days. For the C group, the purified water was administrated by an intragastric dose of 20 ml·kg^-1^ at once a day for 30 days.

### 2.4 Routine blood tests

EDTAK2 anticoagulant whole blood was taken for routine blood test. The routine blood tests were carried out by using Sysmex XN-1000v (B1) automated hematology analyzer (SYSMEX Co., Ltd., Japan).

### 2.5 Detection of CD4^+^ and CD8^+^ T lymphocytes

The positive rates of CD4^+^ and CD8^+^ T lymphocyte subsets were analyzed by flow cytometry. Briefly, 3 ml of red blood cell lysis buffer was added to each sample to completely lyse red blood cells. Then, the samples were washed and re-suspended with DMEM to 1 × 10^6^ cells·ml^-1^. The cells were transferred to 48-well plates with 100 μl volume of each well, and 2 μl of cell activation cocktail (BD Biosciences) and 1 μl of BrefeldinA (BD Biosciences) were added to each sample and incubated at 37°C for 6 h. After washing and re-suspension with phosphate-buffered saline, the samples were incubated with specific fluorescent antibodies (BD Biosciences) of PerCP-Cy™5.5 Hamster Anti-Mouse CD3e (0.5 μl/sample), FITC Rat Anti-Mouse CD4 (0.2 μl/sample), and APC-H7 Rat anti-Mouse CD8a (0.5 μl/sample) for 30 min at room temperature in the dark according to the manufacturer’s guidelines. All samples were stained in triplicate. The samples were analyzed by using the CytoFLEX flow cytometer (Beckman Coulter Life Sciences), and the data were analyzed by the CytExpert software (Beckman Coulter Life Sciences).

### 2.6 Detection of CD4^+^IFN-γ^+^ T lymphocytes

To further determine the levels of CD4^+^IFN-γ^+^ T lymphocyte subsets, the cells treated in the previous step were fixed with 500 μl of 4% FA for 20 min in the dark and ruptured with 1 ml of Permeabilization Wash Buffer (BD Biosciences). Then, the cells were stained with PE Rat Anti-Mouse IFN-γ (1.25 μg/sample) for 30 min. All samples were stained in triplicate. The samples were analyzed by using the CytoFLEX flow cytometer (Beckman Coulter Life Sciences), and the data were analyzed by the CytExpert software (Beckman Coulter Life Sciences).

### 2.7 Serum biochemical analysis

Serum biochemical analysis was detected by using the BS-800 automatic biochemistry analyzer (Shenzhen Mindray Bio-Medical Electronics Co., Ltd., China).

### 2.8 Measurement of serum proteome using DIA-MS

For each sample, 40 ul serum was used for the proteomics analysis. Briefly, total protein was extracted, and albumin/IgG was removed. The protein concentration was determined by the BCA method. Moreover, 10 μg protein of each sample was separated by 12% SDS-PAGE. The digested peptides were desalted by C18-Reverse-Phase SPE Column. RP separation was performed on an 1100 HPLC System (Agilent) using an Agilent Zorbax Extend RP column (5 μm, 150 mm × 2.1 mm). The separated peptides were lyophilized for mass spectrometry. All analyses were performed by a Q-Exactive HF mass spectrometer (Thermo, USA) equipped with a Nanospray Flex source (Thermo, USA). The machine signal is transformed into peptide and protein sequence information by matching the mass spectrum output with the theoretical spectrum generated by fasta library, and then the spectrum Data-Dependent Acquisition library is established by combining the sequence information, peptide retention time, and fragment ion information so as to facilitate the subsequent Data Independent Acquisition (DIA) analysis. The original LC–MS/MS files are imported into the Spectronaut Pulsar software to search and build the database. The original data of DIA is processed by the Spectronaut Pulsar software.

### 2.9 Measurement of serum metabolome and RSGB constituent using nontargeted LC–MS

For each sample, 120 μl serum was added to a 1.5-ml Eppendorf tube with internal standard, and then an ice-cold mixture of methanol and acetonitrile (2:1, v/v) was added to precipitate the protein. The tube was centrifuged, and the final supernatant was filtered through 0.22-μm microfilters and transferred to LC vials. The QC samples were prepared by mixing aliquots of all the 18 samples to be a pooled sample. The vials were stored at 80°C until the LC–MS analysis. An ACQUITY UHPLC system (Waters Corporation, Milford, CT, USA) coupled to an AB SCIEX Triple TOF 5600 System (AB SCIEX, Framingham, MA, USA) was used to analyze the metabolic profiles in both ESI-positive and ESI-negative ion modes. An ACQUITY UPLC BEH C18 column (100 mm × 2.1 mm, 1.7 μm) was employed in both positive and negative modes. The QCs were injected at regular intervals (every six samples) throughout the analytical run to provide a set of data from which repeatability can be assessed. Metabolites were identified by progenesis QI (Waters Corporation, Milford, CT, USA) Data Processing Software, based on public databases such as Human Metabolome Database (http://www.hmdb.ca/), LIPID MAPS Structure Database (http://www.lipidmaps.org/), and METLIN (http://metlin.scripps.edu/index.php).

### 2.10 Measurement of fecal metabolome using nontargeted LC–MS

Fecal sample at 60 mg each was added to a 1.5-ml Eppendorf tube with internal standard, and then an ice-cold mixture of methanol and water (4:1 v/v) was added to each sample. The samples were stored at -20°C for 5 min and then ground at 60 HZ for 2 min, ultrasonicated in ice water bath for 10 min, and stored at -20°C for 30 min. The extract was centrifuged at 13,000 rpm and 4°C for 15 min. Next, 300 μl of supernatant in a brown and glass vial was dried in a freeze concentration centrifugal dryer. Then, a 400-μl mixture of methanol and water (1:4, v/v) was added to each sample; the samples were vortexed for 30 s and then placed at -20°C for 2 h. The samples were centrifuged at 13,000 rpm and 4°C for 10 min. The supernatants (150 μl) from each tube were collected using crystal syringes, filtered through 0.22-μm microfilters, and transferred to LC vials. The vials were stored at -80°C until LC–MS analysis. The QC samples were prepared by mixing aliquots of all the 18 samples to be a pooled sample. A Dionex Ultimate 3000 RS UHPLC system fitted with Q Exactive quadrupole Orbitrap mass spectrometer equipped with a heated electrospray ionization (ESI) source (Thermo Fisher Scientific, Waltham, MA, USA) was used to analyze the metabolic profile in both ESI-positive and ESI-negative ion modes. An ACQUITY UPLC HSS T3 column (100 mm × 2.1mm, 1.8 μm) was employed in both positive and negative modes. The QCs were injected at regular intervals (every six samples) throughout the analytical run to provide a set of data from which repeatability can be assessed. Metabolites were identified by progenesis QI (Waters Corporation, Milford, CT, USA) Data Processing Software based on public databases such as the Human Metabolome Database (http://www.hmdb.ca/).

### 2.11 Measurement of gut microbiota using 16s rDNASeq

Total genomic DNA was extracted using DNA Extraction Kit following the manufacturer’s instructions. The quality and the quantity of DNA was verified with NanoDrop and agarose gel. The extracted DNA was diluted to a concentration of 1 ng·μl^-1^ and was used as the template for the PCR amplification of bacterial 16S rRNA genes with the barcoded primers and Takara Ex Taq (Takara). The V3–V4 variable regions of 16S rRNA genes were amplified with universal primers 343 F and 798 R. The amplicon quality was visualized using gel electrophoresis, purified with AMPure XP beads (Agencourt), and amplified for another round of PCR. After having been purified with the AMPure XP beads again, the final amplicon was quantified using Qubit dsDNA assay kit. Equal amounts of purified amplicon were pooled for subsequent sequencing.

### 2.12 Measurement of plant metabolome in RSGB oral liquid and drug-containing serum using LC–MS/MS

After mixing, 400 ul RSGB oral liquid (R) was taken and was added to a 1.5-ml Eppendorf tube with internal standard. The tube was rolled for 3 min and then centrifuged (10,000 g·min^-1^, 4°C) for 10 min. The supernatant was filtered with a microporous filter membrane (0.22 m) and stored in a sample flask for the LC–MS/MS test. The serum in the RSGB treatment group (L) was thawed on ice, whirled for around 10 s, and then centrifuged at 10,000 g and 4°C for 5 min. Moreover, 50 ul of one sample was taken and homogenized with 1 ml mixture (including methanol, MTBE, and internal standard mixture). The mixture was whirled for 15 min. Then, 200 ul of water was added and whirled for 1 min, and then the mixture was centrifuged at 10,000 g and 4°C for 10 min. The supernatant was extracted and concentrated. The powder was dissolved with 200 ul mobile phase B and then stored at -80°C. Finally, the dissolving solution was put into the sample bottle for the LC–MS/MS analysis.

The sample extracts were analyzed using an UPLC-ESI-MS/MS system (UPLC, SHIMADZU Nexera X2, www.shimadzu.com.cn/; MS, Applied Biosystems 4500 Q TRAP, www.appliedbiosystems.com.cn/). The analytical conditions were as follows: UPLC: column, Agilent SB-C18 (1.8 µm, 2.1 mm × 100 mm). The effluent was alternatively connected to an ESI-triple quadrupole-linear ion trap–MS. LIT and triple quadrupole (QQQ) scans were acquired on a triple quadrupole-linear ion trap mass spectrometer (Q TRAP), AB4500 Q TRAP UPLC/MS/MS System, equipped with an ESI Turbo Ion-Spray interface, operated in positive and negative ion modes, and controlled by Analyst 1.6.3 software (AB Sciex). Instrument tuning and mass calibration were performed with 10 and 100 μmol/L polypropylene glycol solutions in QQQ and LIT modes, respectively. The QQQ scans were acquired as MRM experiments with collision gas (nitrogen) set to medium. DP and CE for individual MRM transitions were done with further DP and CE optimization. A specific set of MRM transitions was monitored for each period according to the metabolites eluted within this period.

### 2.13 Statistical and bioinformatic analysis

All experimental data obtained from rats were expressed as mean ± SD. A one-way repeated-measure analysis of variance and a log-rank test were used to determine the significance of the differences in differential blood count, biochemical index, subsets, and function of lymphocytes, respectively.

For DIA proteomics data, statistical analyses and plots were performed using R Studio software version 1.1.463 (RStudio, Inc.). The database was used to retrieve the original data and keep any set of samples with proteins whose expression value accounts for≥50%. The protein with a missing value <50% is filled in with the half the minimum value of the same group of samples, and the credible protein is obtained through median normalization and log_2_ conversion. Principal component analysis was performed using the expression of the trusted protein. Based on the trusted protein, two standards are selected to calculate the difference between samples. Among them, fold change is used to evaluate the change in the expression level of a certain protein between samples. The *p*-value calculated by the *T*-test shows the significance of the difference between samples. The difference screening conditions were as follows: fold change (FC) =1.2 and *p*-value <0.05. FC >1.2 was considered to be upregulated proteins, while FC <1/1.2 was considered to be downregulated ones. R package was used for the bioinformatics analysis of differentially expressed proteins (DEPs), and these analyses included Metascape online analysis (https://metascape.org/), String online database (https://string-db.org/), and Kyoto Encyclopedia of Genes and Genomes pathway classification.

For LC–MS, differentially expressed metabolites (DEMs) were selected on the basis of the combination of a statistically significant threshold of variable influence on projection (VIP) values obtained from the (orthogonal) partial least-squares-discriminant analysis (OPLS-DA) model and *p*-values from a two-tailed Student’s *t*-test on the normalized peak areas from different groups, where metabolites with VIP values larger than 1.0 and *p*-values less than 0.05 were considered as differential metabolites.

For 16s rDNASeq, the raw sequencing data were in FASTQ format. Paired-end reads were then preprocessed using Trimmomatic software to detect and cut off ambiguous bases (N). It also cut off low-quality sequences with average quality score below 20 using the sliding window trimming approach. After trimming, the paired-end reads were assembled using FLASH software. The parameters of the assembly were as follows: 10 bp of minimal overlapping, 200 bp of maximum overlapping, and 20% of maximum mismatch rate. Sequences were performed upon further denoising as follows: reads with ambiguous homologous sequences or below 200 bp were abandoned, and reads with 75% of bases above Q20 were retained. Then, reads with chimera were detected and removed. These two steps were achieved using QIIME software (version 1.8.0). Clean reads were subjected to primer sequence removal and clustering to generate operational taxonomic units (OTUs) using Vsearch software with 97% similarity cutoff. The representative read of each OTU was selected using the QIIME package. All representative reads were annotated and blasted against Silva database Version 123 (or Greengens) (16s rDNA) using RDP classifier (confidence threshold, 70%). All representative reads were annotated and blasted against Unite database (ITSs rDNA) using blast.

## 3 Results

### 3.1 RSGB reversed agranulocytosis in thyrotoxicosis mice

**Figure 2:**
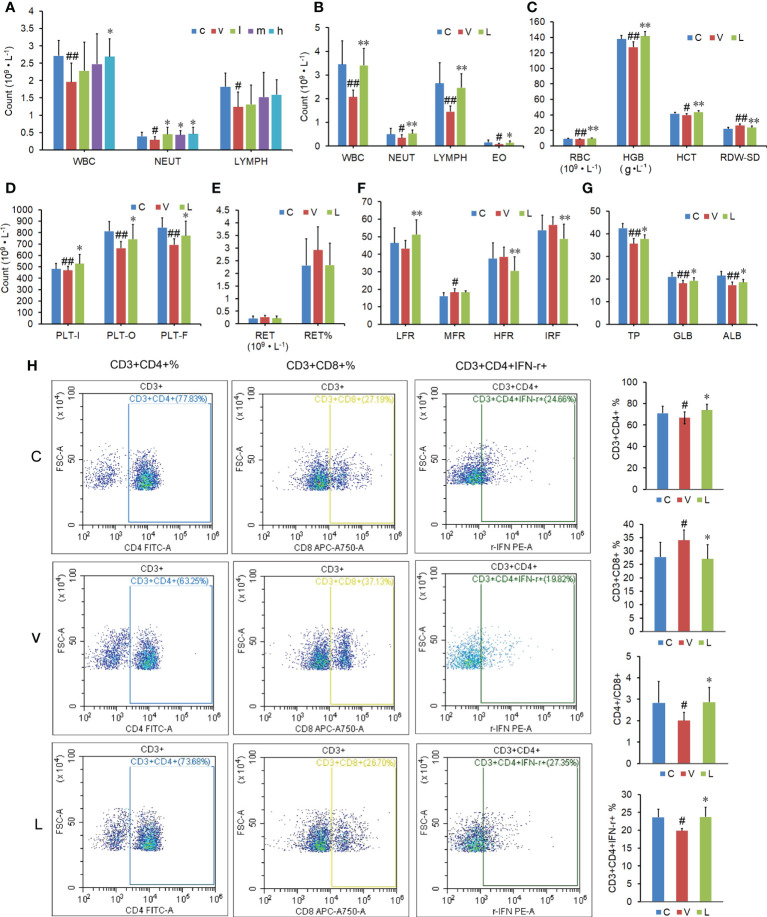
Effect of Renshen Guben (RSGB) on differential blood count, subsets and function of lymphocytes, and biochemical index in thyrotoxicosis mice. **(A)** The RSGB treatment (5, 10, and 20 ml·kg^-1^, once per day, 20 days) significantly increased the neutrophils. **(B)** The RSGB treatment (5 ml·kg^-1^, twice per day, 30 days) significantly increased the white blood cells, neutrophils, lymphocytes, and eosinophils in thyrotoxicosis mice. **(C)** The RSGB treatment recovered red blood cells (RBC), hemoglobin, hematocrit, and RDW-SD, which indicated that RSGB could improve anemia in thyrotoxicosis mice. **(D)** The RSGB treatment increased the count of platelets. **(E, F)** The reticulocyte count indicated that the RSGB treatment promoted RBC maturation and alleviated anemia in thyrotoxicosis mice. **(G)** The RSGB treatment significantly increased the levels of total protein, albumin, and globulin in thyrotoxicosis mice, which indicated that the RSGB treatment improved the hypoproteinemia in thyrotoxicosis mice. **(H)** The RSGB treatment increased the proportion of CD4+/CD8+ and percent CD3+CD4+ IFN-**γ**+, which indicated that RSGB improved the immunosenescence in thyrotoxicosis mice. Data are mean ± SD, *n* = 6; ^#^ *p* < 0.05 and ^##^ *p* < 0.01 *vs*. normal control mice; ^*^ *p* < 0.05 and ^**^ *p <*0.01 *vs*. vehicle treatment mice. c, normal control group; v, vehicle treatment group; l, m, h, RSGB treatment (5, 10, and 20 ml·kg^-1^) groups; C, normal control group; V, vehicle treatment group; L, RSGB treatment (5 ml·kg^-1^) group.

The blood routine test results showed that the white blood cells (WBC), neutrophils (NEUT), and lymphocytes (LYMPH) were significantly decreased after a 10-day overdose of thyroxine. Only a high dose of RSGB (20 ml·kg^-1^) increased the WBC in in thyrotoxicosis mice, while all three doses of RSGB (5, 10, and 20 ml·kg^-1^ at once per day for 20 days) increased the NEUT in thyrotoxicosis mice (**Figure 2A**), which suggested that RSGB could reverse agranulocytosis in thyrotoxicosis mice. LYMPH also showed an increasing trend in the RSGB treatment group, so the following study prolonged the administration frequency and time.

### 3.2 RSGB alleviated leukocytopenia and anemia in thyrotoxicosis mice

The blood routine test results showed that the whole blood cells were significantly decreased after a 20-day overdose of thyroxine. The blood routine test results showed that the decreased WBC, NEUT, LYMPH, and eosinophils in thyrotoxicosis mice were recovered by RSGB treatment (5 ml·kg^-1^ twice per day for 30 days) (**Figure 2B**), which indicated that RSGB reversed leukocytopenia in thyrotoxicosis mice. Red blood cells (RBC), hemoglobin (HGB), hematocrit (HCT), and RDW-SD were also increased in the RSGB treatment group (**Figure 2C**), which indicated that RSGB alleviated anemia in thyrotoxicosis mice. The count of platelets (PLTs) was also increased in the RSGB treatment group (**Figure 2D**), which suggested that RSGB could prevent thrombocytopenia in thyrotoxicosis mice. Although the difference in reticulocyte count between the RSGB-treated mice and the thyrotoxicosis mice was not significant, the trend was increasing (**Figure 2E**). Moreover, the low-fluorescence-intensity reticulocyte was increased, while the high-fluorescence-intensity reticulocyte was decreased and the immature reticulocyte fraction was decreased in the RSGB treatment group, which indicated that the RSGB treatment promoted RBC maturation and alleviated anemia in thyrotoxicosis mice (**Figure 2F**).

### 3.3 RSGB improved hypoproteinemia in thyrotoxicosis mice

The biochemical test showed that the levels of total protein (TP), albumin, and globulin were significantly increased in the RSGB treatment group, which indicated that RSGB treatment improved hypoproteinemia and hypoalbuminemia in thyrotoxicosis mice (**Figure 2G**).

### 3.4 RSGB enhanced the function of lymphocytes in thyrotoxicosis mice

The results of flow cytometry showed that the RSGB treatment significantly increased the ratio of CD4+/CD8+ and the percent CD3+CD4+ IFN-**γ**+ in thyrotoxicosis mice (**Figure 2H**). It indicated that RSGB improved the immunosenescence and recovered the function of lymphocytes.

### 3.5 Comparative serum proteomic analysis

**Figure 3:**
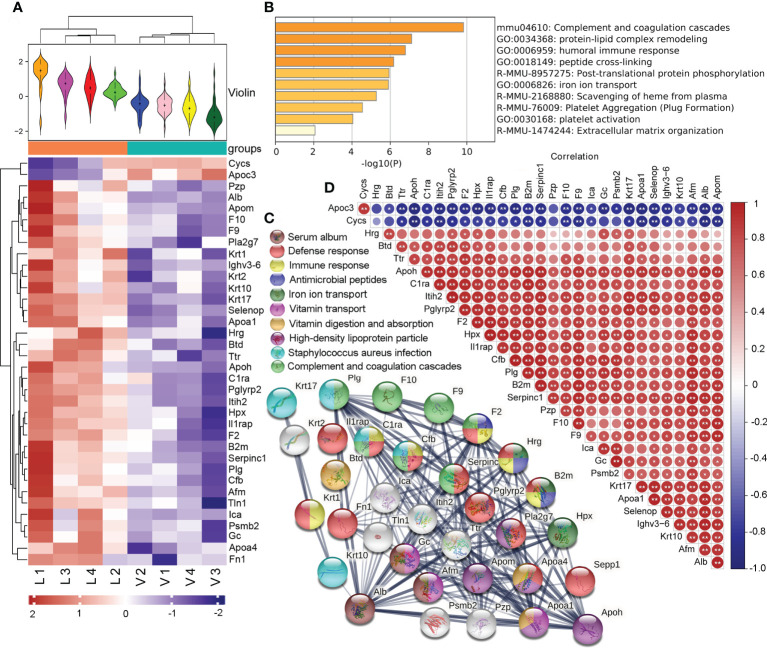
Effect of Renshen Guben (RSGB) on serum proteome in thyrotoxicosis mice. **(A)** There were 36 differentially expressed proteins (DEPs) between the thyrotoxicosis mice (V) and the RSGB-treated mice (L): two were downregulated and 34 were upregulated. **(B)** A total of 34 upregulated proteins were analyzed by Metascape online analysis. **(C)** The STRING online analysis (https://string-db.org/) displayed the clusters of upregulated DEPs. **(D)** Correlation analysis of DEPs. Red is positive correlation, and blue is negative correlation; correlation significance: ^*^ *p* < 0.05, ^**^ *p* < 0.01. V, vehicle treatment group; L, RSGB treatment (5 ml·kg^-1^) group.

Using the expression of believable protein for principal component analysis, there was a significant difference between the thyrotoxicosis mice (V) and the RSGB-treated mice (L), the proteomic analysis highlighted 36 DEPs between the two groups; among them, 2 were downregulated and 34 were upregulated (**Figure 3A**). The two downregulated proteins were Cycs and Apoc3. The 34 upregulated proteins were analyzed by Metascape online analysis. The enrichment terms were as follows: complement and coagulation cascades, protein–lipid complex remodeling, humoral immune response, peptide cross-linking, post-translating protein phosphorylation, iron ion transport, scavenging of heme from plasma, platelet aggregation, platelet activation, and extracellular matrix organization (**Figure 3B**).

The STRING online analysis (https://string-db.org/) displayed the details of the upregulated DEPs (**Figure 3C**). The enrichment clusters were highlighted and marked with different colors, namely: serum album (brown), defense response (red), immune response (light yellow), antimicrobial peptides (dark blue), iron ion transport (dark green), vitamin transport (light purple), vitamin digestion and absorption (dark yellow), high-density lipoprotein particle (dark purple), *Staphylococcus aureus* infection (light blue), and complement and coagulation cascades (light green). Of these upregulated proteins, 12 were related to defense response, 10 were related to immune response, and three were antimicrobial peptides, which suggested that defense ability was increased by the RSGB treatment.

The correlation analysis showed that the downregulated DEPs Apoc3 and Cycs were negatively correlative with most of the upregulated DEPs. The Alb was positively correlative with most of the upregulated DEPs (**Figure 3D**).

The upregulation of Alb was in concordance with the result of biochemistry, which indicated that RSGB treatment significantly improved the hypoalbuminemia in thyrotoxicosis mice. The upregulation of Ighv3-6 was in line with the result of Glb in the biochemistry test, which indicated that the immunoglobin or antibody was increased by the RSGB treatment. The upregulation of DEPs that accounted for lipid transport, ion transport, and vitamin transport indicated that the usage of lipid, ions, and vitamins was improved in the RSGB-treated mice. In summary, the results of serum proteomics showed that the RSGB treatment significantly improved the defense ability and malnutrition in thyrotoxicosis mice.

### 3.6 LC–MS metabolomics analysis in serum and feces

**Figure 4:**
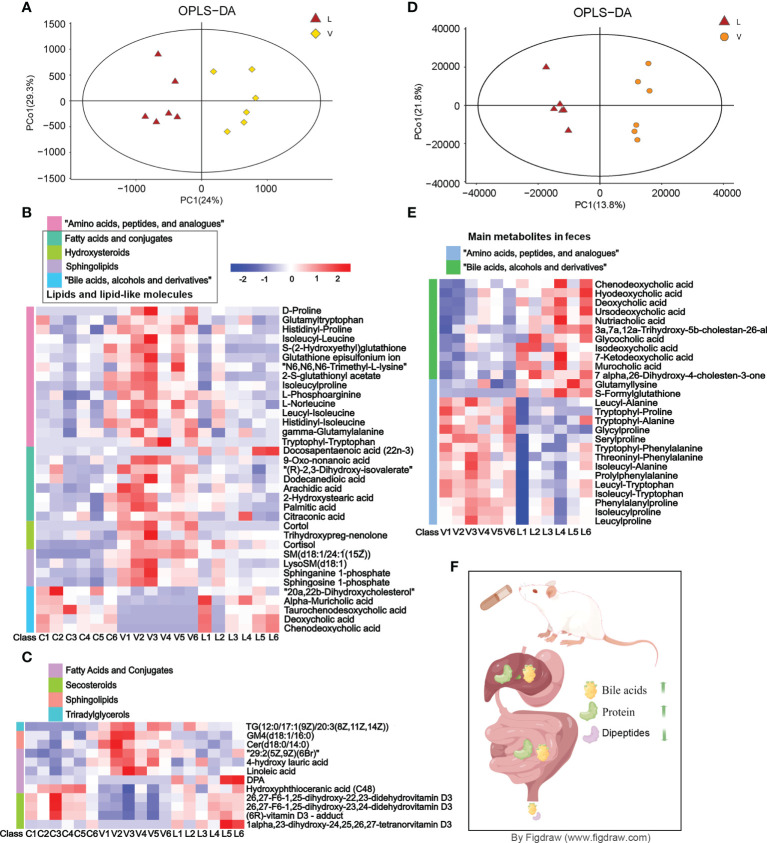
Effect of Renshen Guben (RSGB) on the metabolome of serum and feces in thyrotoxicosis mice. **(A)** The serum metabolomics analysis result showed that there were significant differences in the OPLS-DA score between the RSGB treatment group (L) and the vehicle treatment group (V). **(B)** The heat map shows that the serum differentially expressed proteins (DEPs) in amino acids, peptides, and analog classes were downregulated in the RSGB treatment group. All hydroxysteroids, sphingolipids and most of the fatty acids were downregulated, whereas all bile acids, alcohols, and derivatives were upregulated and docosapentaenoic acid (DPA) was upregulated in the RSGB treatment group. **(C)** The heat map shows the results of lipomics in serum: TG[12:0/17:1(9Z)/20:3(8Z,11Z,14Z)], GM4(d18:1/16:0), Cer(d18:0/14:0), 29:2(5Z,9Z)(6Br), 4-hydroxy lauric acid, and linoleic acid were downregulated, while DPA, hydroxyphthioceranic acid (C48), 26,27-F6-1,25-dihydroxy-22,23-didehydrovitamin D3, 26,27-F6-1,25-dihydroxy-23,24-didehydrovitamin D3, (6R)-vitamin D3-adduct, and 1alpha,23-dihydroxy-24,25,26,27-tetranorvitamin D3 were upregulated. **(D)** The feces metabolomics analysis result showed that there were significant differences in the OPLS-DA score between the RSGB treatment group (L) and the vehicle treatment group (V). **(E)** Main differentially expressed metabolites in feces. The class of amino acids, peptides, and analogs were downregulated, while the class of bile acids was significantly upregulated in the feces of RSGB-treated mice. **(F)** Schematic diagram of bile acid, amino acid, and protein anabolism. The RSGB oral liquid treatment increased the liver synthesis and the intestinal transformation of bile acids, so the excretion of bile acids in feces increased. The protein synthesis in the liver and the absorption in the intestine were increased, while the loss of amino acids and peptides in feces was significantly reduced. Therefore, hypoproteinemia was improved.

The serum metabolomics analysis result showed that there were significant differences in the OPLS-DA score between the RSGB treatment group (L) and the thyrotoxicosis group (V) (**Figure 4A**). Based on Human Metabolome Database (HMDB), compared with vehicle treatment thyrotoxicosis mice, 62 DEMs were screened in the serum of RSGB-treated mice, including 19 upregulated and 43 downregulated (**Supplementary Table S1**). The heat map showed the abundance of some DEMs (**Figure 4B**). The DEMs in amino acids, peptides, and analogues class were downregulated, which indicated that the decomposition of protein in tissue was significantly inhibited. The DEMs in the class of lipids and lipid-like molecules included several subclasses, among them are hydroxysteroids, sphingolipids, and most fatty acids which were downregulated, while all bile acids, alcohols, and derivatives were upregulated; DPA was also upregulated.

Based on the Lipidmaps (v2.3) database, compared with vehicle treatment thyrotoxicosis mice, LC–MS-based metabolomics screened 163 lipid DEPs in the serum of RSGB-treated mice, including 97 upregulated and 66 downregulated (**Supplementary Table S2**). The heat map shows the details of some DEMs in each mouse (**Figure 3B**). TG [12:0/17:1(9Z)/20:3(8Z,11Z,14Z)], GM4 (d18:1/16:0), Cer (d18:0/14:0), 29:2(5Z,9Z)(6Br), 4-hydroxy lauric acid, and linoleic acid were downregulated, while DPA, hydroxyphthioceranic acid (C48), 26,27-F6-1,25-dihydroxy-22,23-didehydrovitamin D3, 26,27-F6-1,25-dihydroxy-23,24-didehydrovitamin D3, 1alpha,23-dihydroxy-24,25,26,27-tetranorvitamin D3, and (6R)-vitamin D3-adduct were upregulated (**Figure 4C**).

The fecal metabolomics analysis result showed that there were significant differences in OPLS-DA score between the RSGB treatment group (L) and the thyrotoxicosis group (V) (**Figure 4D**). Based on HMDB, 62 DEMs were screened in feces, including 35 upregulated and 27 downregulated (**Supplementary Table S3**). The class of amino acids, peptides, and analogues was the largest, and most of them were downregulated. The class of bile acids was significantly upregulated in the feces of the RSGB-treated mice (**Figure 4E**). The schematic diagram of bile acid, amino acid, and protein anabolism is shown in **Figure 4F**. For bile acids, RSGB oral liquid treatment increased liver synthesis and intestinal transformation, so fecal excretion was increased. For protein and amino acids, liver protein synthesis increased, and intestinal absorption improved. The loss of amino acids and peptides in feces was reduced; therefore, hypoproteinemia was improved.

### 3.7 Changes of microbiome and its correlation with metabolites of RSGB in feces

**Figure 5:**
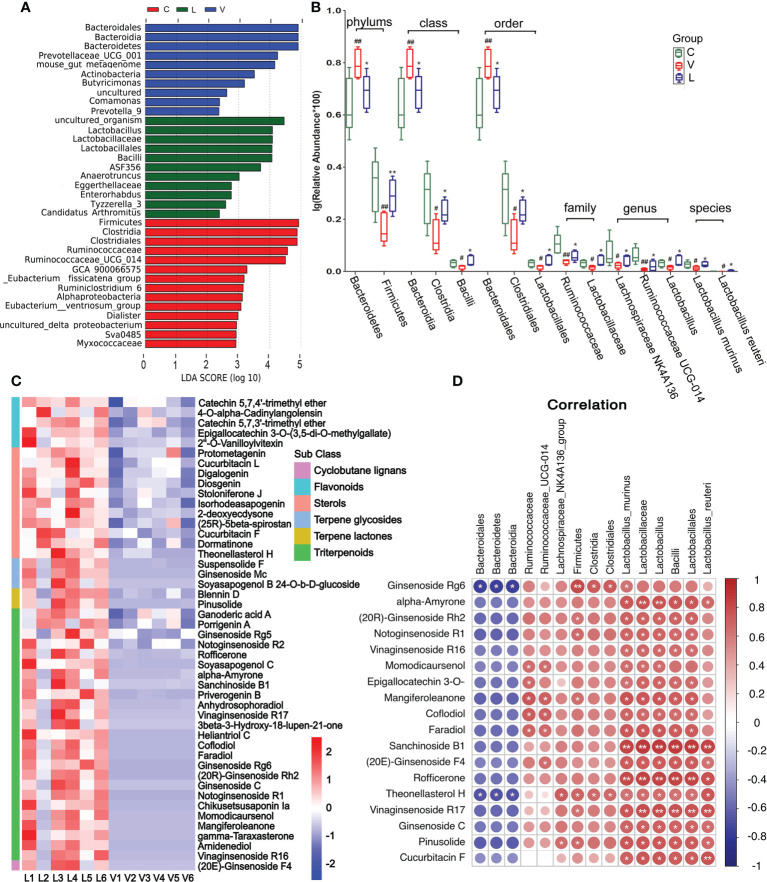
Effect of Renshen Guben (RSGB) on microbiome in feces in thyrotoxicosis mice and the correlation of microbiome with RSGB metabolites. **(A)** LEfSe showed species with relatively high abundance in each group. **(B)** Microbe abundance with significant differences at the phylum, class, order, family, genus, and species levels. #p < 0.05 and ##p < 0.01 vs. normal control mice; *p < 0.05 and **p < 0.01 vs. vehicle treatment mice. **(C)** RSGB metabolites in the feces of the RSGB-treated mice. **(D)** The correlation of microbiome with RSGB metabolites in feces in the RSGB treatment group, red is positive correlation, and blue is negative correlation; correlation significance: *p < 0.05, **p < 0.01. C, normal control group; V, vehicle treatment group; L, RSGB treatment (5 ml·kg^-1^) group.

LEfSe showed species with relatively high abundance in each group (**Figure 5A**). The score map showed the abundance of *Firmicutes*, *Clostridia*, *Clostridiales*, *Ruminococcaceae*, *Ruminococcaceae_UCG_014*, *GCA_900066575*, *_Eubacterium:fissicatena_group*, *Ruminiclostridium_6*, *Alphaproteobacteria*, *_Eubacterium:ventriosum_group*, *Dialister* were high in the normal control group. The abundance of *Bacteroidales*, *Bacteroidia*, *Bacteroidetes*, *Prevotellaceae_UCG_001*, *mouse_gut_metagenome*, *Actinobacteria*, *Butyricimonas*, *Comamonas*, and *Prevotella_9* were high in the vehicle treatment group. *Lactobacillus*, *Lactobacillaceae*, *Lactobacillales*, *Bacilli*, *ASF356*, *Anaerotruncus*, *Eggerthellaceae*, *Enterorhabdus*, *Tyzzerella_3*, and *Candidatus_Arthromitus* were high in the RSGB treatment group.

Microbe abundance with significant differences at the phylum, class, order, family, genus, and species levels are shown in **Figure 5B**. Compared with the normal control group, at the phylum level, the abundance of *Bacteroidetes* was significantly increased, while that of *Firmicutes* was decreased in the vehicle treatment group. At the class level, the abundance of *Bacteroidia* was significantly increased, while those of *Clostridia and Bacilli* were significantly decreased. At the order level, *Bacteroidales* was significantly increased, while *Clostridiales* and *Lactobacillales* were significantly decreased. At the family level, *Ruminococcaceae* and *Lactobacillaceae* were significantly decreased. At the genus level, *Lachnospiraceae_NK4A136*, *Ruminococcaceae_UCG-014*, and *Lactobacillus* were significantly decreased. At the species level, *Lactobacillus_murinus* and *Lactobacillus_reuteri* were significantly decreased. Compared with the vehicle treatment group, all the above-cited microbe abundances were reversed in the RSGB treatment group.

A large number of RSGB metabolites were detected in the feces of the RSGB-treated group, including triterpenoids, terpene lactones, terpene glycosides, sterols, flavonoids, and cyclobutane lignans (**Figure 5C**). The correlation analysis showed that several RSGB metabolites were positively with gut microbe, especially ginseng metabolites and *Lactobacillales* (**Figure 5D**).

### 3.8 The safety of RSGB oral liquid

The safety of 30 days of treatment of RSGB oral liquid (20 ml·kg^-1^, W) was investigated. Compared with normal control mice (N), only TG and LDL-C were found decreased in biochemical tests in the RSGB treatment alone group. WBC, NEUT, and LYMPH were slightly elevated in the routine blood test, but within normal range. All these changes might be the embodiment of a pharmacodynamic effect. The rest indexes were normal. Pathological examination showed no obvious pathological changes. It indicated that the long-term administration of RSGB oral liquid for 30 days had no toxic reactions, which proved the safety of RSGB oral liquid. The results are listed in **Table 1**.

**Table 1:** Blood routine and biochemical indicators.

		N (n = 9)	W (n = 9)
Routine blood test			
WBC	10^9^/L	2.78 ± 0.41	3.78 ± 1.23^*^
RBC	10^12^/L	8.93 ± 1.11	8.92 ± 0.89
HGB	g/L	133.13 ± 14.87	130.00 ± 16.01
HCT	%	44.13 ± 4.32	43.11 ± 4.41
MCV		247.88 ± 9.72	241.61 ± 7.03
MCH		74.63 ± 2.40	72.72 ± 3.78
MCHC		1,506.25 ± 27.48	1,505.56 ± 48.89
PLT	10^9^/L	1,027.50 ± 117.29	1,006.67 ± 188.63
RDW-SD		25.18 ± 1.61	24.39 ± 1.63
RDW-CV		12.99 ± 0.59	12.91 ± 0.52
PDW		5.00 ± 0.16	4.97 ± 0.26
MPV		34.56 ± 0.82	34.17 ± 0.97
P-LCR	%	14.50 ± 5.36	10.89 ± 4.92
PCT		0.38 ± 0.05	0.36 ± 0.07
NEUT	10^9^/L	0.39 ± 0.13	0.61 ± 0.16^**^
LYMPH	10^9^/L	1.91 ± 0.33	2.64 ± 1.26^*^
MONO	10^9^/L	0.27 ± 0.11	0.29 ± 0.08
EO	10^9^/L	0.21 ± 0.18	0.14 ± 0.07
Biochemical test			
T-Bil-V	μmol/L	0.43 ± 0.39	0.58 ± 0.48
ALT	U/L	36.83 ± 6.97	37.13 ± 9.14
AST	U/L	81.56 ± 10.47	83.56 ± 19.90
TP	g/L	49.78 ± 2.21	48.67 ± 2.29
TG	mmol/L	1.86 ± 0.40	1.17 ± 0.25^**^
LDL-C	mmol/L	0.21 ± 0.07	0.16 ± 0.03^*^
HDL-C	mmol/L	1.97 ± 0.43	1.80 ± 0.22
TC	mmol/L	2.38 ± 0.54	2.07 ± 0.26
UREA	mmol/L	8.61 ± 1.23	9.89 ± 1.21
LDH	U/L	1,111.89 ± 145.87	1,114.28 ± 506.66
ALB II	g/L	24.44 ± 1.38	23.94 ± 1.57
CREA-S	μmol/L	23.72 ± 0.97	23.50 ± 1.25

Compared with normal control mice, *p< 0.05; **p< 0.01.

N, normal control group; W, RSGB group (20 ml·kg^-1^).

### 3.9 Active ingredient in RSGB oral liquid and drug-containing serum

**Figure 6:**
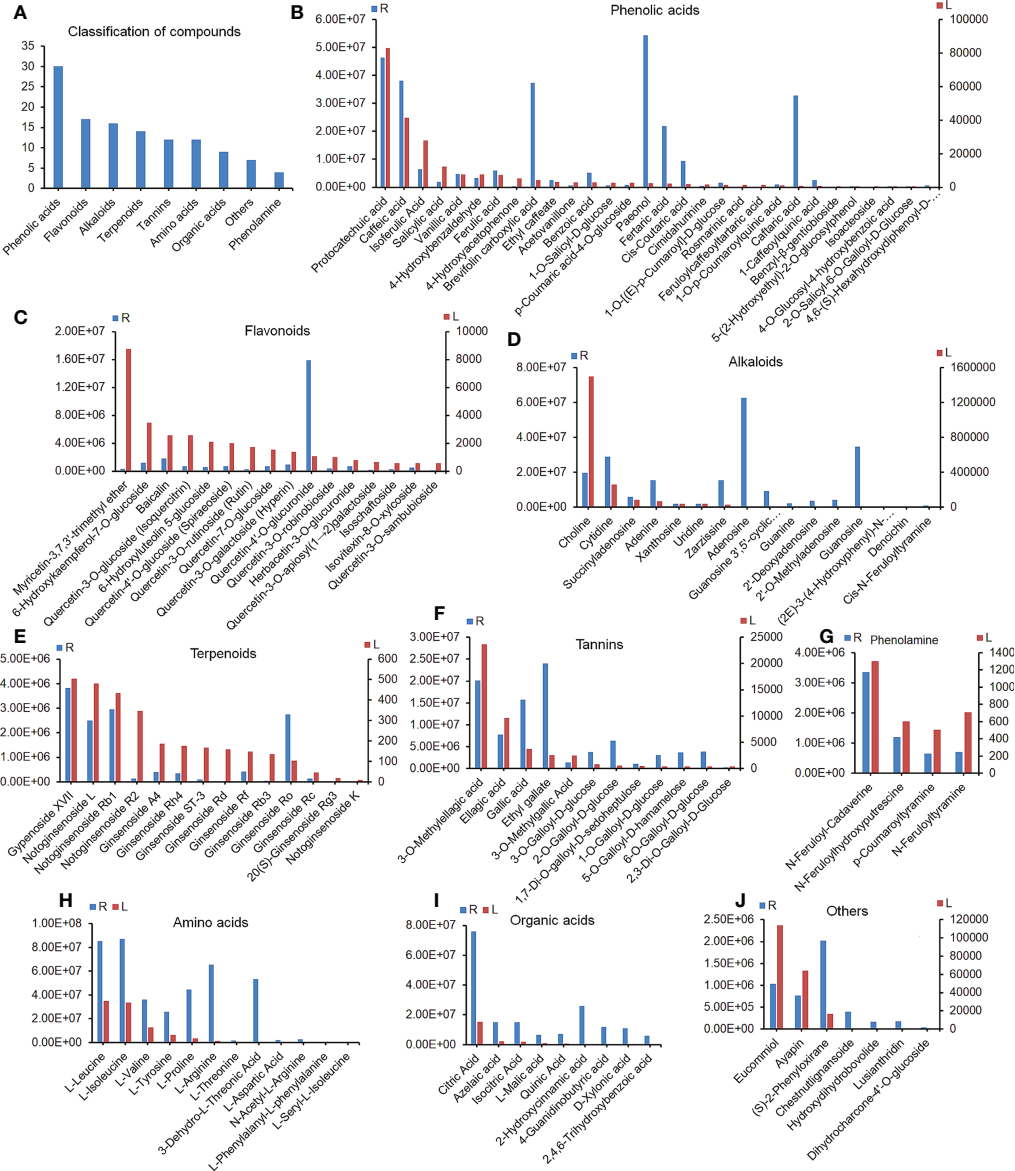
Constituents in Renshen Guben (RSGB) oral liquid and drug-containing serum. A total of 121 plant components were identified in RSGB oral liquid (R) and drug-containing serum (L) by plant metabonomics **(A)**, including 30 phenolic acids **(B)**, 17 flavonoids **(C)**, 16 alkaloids **(D)**, 14 terpenoids **(E)**, 12 tannins **(F)**, four phenolamines **(G)**, 12 amino acids **(H)**, nine organic acids **(J)**, and seven others **(I)**. They were the material basis of the pharmacological action of RSGB oral liquid (*n* = 3).

The plant metabolome showed that a total of 121 plant components were identified and quantified by LC–MS/MS in RSGB oral liquid (R) and drug-containing serum (L), including 30 phenolic acids,16 flavonoids, 16 alkaloids, 14 terpenoids, 12 amino acids, nine organic acids, 12 tannins, four phenolamines, and seven others (**Figure 6A**). The phenolic acids that were present at higher levels in both drug and medicated serum included protocatechuic acid, caffeic acid, isoferulic acid, salicylic acid, vanillic acid, 4-hydroxybenzaldehyde, and ferulic acid (**Figure 6B**). Myricetin-3,7,3’-trimethyl ether was one of the most abundant flavonoids in medicated serum. 6-Hydroxykaempferol-7-O-glucoside, baicalin, and many kinds of quercetin derivatives were detected in both RSGB oral liquid and medicated serum (**Figure 6C**). Alkaloids included choline and many nucleosides, such as cytidine, succinyladenosine, adenine, xanthosine, uridine, zarzissine, adenosine, guanosine 3’,5’-cyclic monophosphate, guanine, 2’-deoxyadenosine, 2’-O-methyladenosine, and guanosine (**Figure 6D**). Terpenoids included gypenoside XVII, notoginsenoside L, notoginsenoside Rb1, notoginsenoside R2, ginsenoside A4, ginsenoside Rh4, ginsenoside ST-3, ginsenoside Rd, ginsenoside Rf, ginsenoside Rb3, ginsenoside Ro, ginsenoside Rc, 20(S)-ginsenoside Rg3, and notoginsenoside K (**Figure 6E**). The tannins were mainly ellagic acid, gallic acid, and their derivatives (**Figure 6F**). There were four phenolamines, N-feruloyl-cadaverine, N-feruloylhydroxyputrescine, p-coumaroyltyramine, and N-feruloyltyramine (**Figure 6G**). There were many amino acids and organic acids in RSGB oral liquid and medicated serum, whereas these nutrients are also endogenous, so this portion of the serum data may not be drug sources only (**Figures 6H, I**). However, it indicated that RSGB oral liquid can directly supply nutrients to the body. Others included eucommiol, ayapin, (S)-2-phenyloxirane, and so on. The abundance of eucommiol and ayapin was relatively higher in medicated serum (**Figure 6J**). These substances should be the material basis of the pharmacological action of RSGB oral liquid.

## 4 Discussion

With increasing age, the immune function of the body gradually decreases, leading to susceptibility to infectious diseases, tumors, autoimmune diseases, and some geriatric diseases. In addition to immunity, aging manifests itself in various aspects such as physiology, biochemistry, metabolism, endocrine, and organs, and all these aspects interacted with each other, so aging is the superposition effect of a series of events. Models of aging that can respond to these events are of great scientific interest, and drugs that have effects on these aspects will have enormous potential for anti-aging. Our previous study showed that the thyrotoxicosis mouse model is one such model (24). In this model, in terms of physiology, the total number of whole blood cells was decreased, the TP and Alb were decreased in biochemistry, and, in terms of immunity, the number and function of immune cells were decreased, and the levels of complement, antimicrobial peptide, and immunoglobulin were decreased. In terms of metabolism, lipotoxicity was increased, and the endogenous glucocorticoids were significantly upregulated on the endocrine side. All these manifestations are associated with aging and aging-related diseases, which were fully discussed in our previous report, and some serum biomarkers of aging in the model were highlighted, such as Alb, prealbumin (Ttr), histidine-rich glycoprotein (Hrg), glucocorticoids, linoleic acid, sphingolipids, VD3, and DPA. The candidates that can improve one or more of these events or biomarkers might have an anti-aging effect. In the present study, the anti-aging effect of a TCM prescription RSGB was confirmed on a thyrotoxicosis mice model, and the anti-aging mechanism has been fully explored.

RSGB significantly improved immunosenescence in thyrotoxicosis mice, which was manifested in increasing the total number of leukocytes, neutrophils, lymphocytes, and the percent of CD4/CD8 and CD3+CD4+ IFN-**γ**+. These were the signs of RSGB improving immunity at the cellular level. At the molecular level, RSGB significantly increased the abundance of complement and coagulation components as well as AMPs. As well known, the complement system is the main component of the immune system (26). The coagulation system also plays an important role in innate immunity (27), which could prevent microbial invasion by forming physical barriers, and some members themselves were AMPs. AMPs are a group of small proteins that act as a first line of defense against invading microbes, so they are also important effectors of innate immunity (28). In the present study, prothrombin (F2), β-2-microglobulin (B2m), and Hrg were the three upregulated AMPs in the RSGB treatment group. These AMPs have both antimicrobial activity and immune regulation function (29–33). B2m and Hrg also have iron transport ability; their absence will lead to severe anemia and tissue iron overload (34). Anemia weakens immunity and increases an individual’s susceptibility to infectious diseases, such as tuberculosis (35). The thyrotoxicosis mice also have anemia symptoms, while RSGB treatment significantly improved anemia. Besides iron, selenium was associated with anemia (36, 37). Selenoprotein P (Sepp1) plays a key role in selenium homeostasis and defense ability (38–40), and it was upregulated in the RSGB treatment group, which might be helpful to maintain the defense ability in thyrotoxicosis mice.

Nutritional status is closely related to resistance. Good nutritional status helps to maintain good constitution for resisting diseases and recovering. In addition to the improvement of anemia, the upregulation of Alb and Ttr also indicated that RSGB improved the nutritional status of the thyrotoxicosis mice. Hypoalbuminemia is a common problem during hospitalization (41). Preoperative Ttr plays an important role in predicting postoperative complications (42). Decreased Alb and Ttr levels were both associated with an increased risk of death in hospitalized elderly COVID-19 patients (43, 44), so it is very important to maintain the protein level in the clinic. However, there are many problems in clinical direct Alb infusion, such as limited effect, large side effects, and high economic burden (45). In the present study, the RSGB treatment significantly increased the TP level in the serum of thyrotoxicosis mice by increasing synthesis, reducing decomposition, and inhibiting loss. It is of great significance for the elevation of the level of Alb and Ttr by orally administrating RSGB, which will be helpful for improving the prognosis of hospitalized patients, especially the elderly. In addition, the transport and usage of micronutrient vitamins was also improved in the RSGB treatment group. The levels of biotinidase, vitamin D-binding protein (VDBP, Gc), and vitamin E-binding protein (Afamin, Afm) were upregulated in the RSGB treatment group, which were beneficial for the usage of biotin, VD, and VE. It was helpful to maintain the normal physiological function of thyrotoxicosis mice. The results of lipid metabolomics showed that 26,27-F6-1,25(OH)2D3 and 1alpha,23-dihydroxy-24,25,26,27-tetranorvitamin D3 were elevated in the RSGB treatment group. They might work together with VDBP to contribute to the protective effect of RSGB on thyrotoxicosis mice. Totally, the upregulation of ion transport proteins, vitamin-binding proteins, VD3, Alb, and Ttr indicated that malnutrition was significantly improved in the RSGB treatment group.

Proteomics and metabonomics together showed the evidence of RSGB-reduced lipotoxicity. VDBP, Alb, α-fetoprotein, and Afm are four members of the albumin gene family. All of them are involved in the binding and transport of fatty acids and protect against free fatty acid toxicity (46). The physiological concentrations of unsaturated fatty acids linoleic or arachidonic acid impaired the binding of VD to VDBP (47), which might be one of the manifestations of unsaturated fatty acid lipid toxicity. The unbound fatty acids, such as linoleic acid, reacted with Alb and induced hypoalbuminemia (48). Therefore, the upregulation of VDBP, Alb, and Afm in the RSGB treatment group also contributed to the reduction of lipotoxicity. Non-esterified fatty acids, such as oleic acid and linoleic acid induced neutrophil extracellular traps (NETs) extrusion and NETosis, disrupt the immune functions and induce organ injury (49, 50). Sphingosine, linoleic acid, and palmitic acid destroyed the mitochondrial function of immune cells by oxidative damage (24). All of them were downregulated by RSGB treatment. As well known, glucocorticoids have an immunosuppressive effect, and stress-induced glucocorticoids might play a causal role in aging and age-related disorders (51). Because glucocorticoids are lipidoid molecules, they can also be classified as lipotoxic molecules, and the downregulation of glucocorticoids was also one of the manifestations of RSGB alleviating lipotoxicity in thyrotoxicosis mice. Apom, Apoa1, Apoa4, and Apoh were high-density lipoprotein particles; their upregulation improved the lipid transport and lipotoxicity (52). The downregulation of Cycs was one evidence for reducing mitochondrial lipotoxicity in RSGB-treated mice (53). More interestingly, the protective fatty acid DPA was significantly increased in the RSGB treatment group, which might be helpful to counter the lipotoxicity. As reported, ω-3 fatty acids could resist the adverse effects of cortisol, enhance cognitive ability, optimize neuromuscular function and reduce muscle loss, regulate lipid metabolism, and improve cardiovascular disease, thus improving the immune status and protecting against infection and allergies; it even has the potential to resist COVID-19, and the protective activity of DPA was better than EPA and DHA (24). DPA can protect older people from disease (54, 55). Therefore, it was of great significance for RSGB to increase the level of DPA.

The RSGB treatment also had a significant impact on the intestinal defense ability by regulating the level of bile acids and intestinal flora. The combination of serum and fecal metabolomics showed the recovery of the bile acid spectrum in the RSGB treatment group. Several bile acids were found upregulated in both serum and feces in RSGB-treated mice. Many kinds of gut microbiota have the ability of transforming primary bile acids to secondary bile acids, such as *Clostridia* and *Lactobacillus*. Bile acid metabolism is regulated by the intestinal flora and maintains the intestinal barrier through a variety of effects, such as antibacterial effect, inhibiting microbial metabolism, promoting mucus production, maintaining epithelial integrity, tight junction regulation, reducing endoplasmic reticulum pressure, inhibiting proinflammatory cytokines, and immune regulation (56). In the present study, the 16S rDNA sequence analysis showed that *Bacilli*, *Clostridiales*, *Lactobacillales*, *Lactobacillaceaem*, *Lactobacillus*, *Lactobacillus_murinus*, and *Lactobacillus_reuteri* were significantly upregulated in the RSGB treatment group. As well known, lactic acid bacteria have many beneficial effects on the host and have the potential to limit the clinical use of antibiotics. *Lactobacillus reuteri* (*L. reuteri*) is a well-studied probiotic bacterium that has a defensive effect on both the local part of the intestine and the whole body (57). *Lactobacillus_murinus* (*L. murinus*) have similar beneficial effects (58, 59). Although the oral supplementation of probiotics is feasible, due to the destructive effect of digestive juice and colonization resistance caused by the original symbiotic bacteria, the number of probiotics reaching the intestine may not reach the effective number (60). The present study proved that the oral administration of TCM such as RSGB can increase the abundance of lactic acid bacteria. As reported, lactic acid bacteria had the ability to increase the plasma concentration of deglycosylated ginsenosides (61). *Lactobacillus reuteri* could glucosylate the acceptor substrates caffeic acid and gallic acid (62). The correlation analysis showed that several RSGB metabolites positively interacted with the probiotics. Therefore, the mutual promotion between LAB and RSGB metabolites in this study contributed to maintaining intestinal health.

Actually, there are many components that can interact with the intestinal flora. In addition to interacting with the intestinal flora, the active components in RSGB also have direct effects. Clarifying these components is very important so as to expand the cognition of TCM. In the present study, UPLC–MS/MS-based plant metabolomics methods were carried out to analyze the molecular composition in both RSGB oral liquid and the serum of RSGB-treated mice. The results showed that there were many kinds of substances in the oral liquid and the serum of RSGB-treated mice, including terpenoids, phenolic acids, flavonoids, tannin, alkaloids, organic acids, phenolamines, amino acids, and so on. These substances might be the active ingredients in the RSGB oral liquid.

Ginseng and its active components have excellent performance in anti-aging, including anti-oxidant, immune regulation, and organ protection properties (14). Ginsenosides were the main active components in ginseng (63). A total of 14 triterpenoid ginsenosides were detected simultaneously in the RSGB oral liquid and drug-containing serum. They have been reported to have excellent biological activities, such as protecting the mitochondria (64, 65), reducing the injury from oxidized LDL and ROS (66), protecting the intestinal barrier by regulating the intestinal microbiota and bile acids (67), enhancing immune response (68–70), and anti-aging (71, 72). All these activities are consistent with the pharmacodynamic effect of RSGB in this study. Therefore, these triterpenoid ginsenosides are important active components of RSGB. Phenolic acids were a large group of components in RSGB oral liquid and drug-containing serum. The representatives of phenolic acids include protocatechuic acid, caffeic acid, and paeonol; they all had a wide range of pharmacological activities, including antioxidant, immunomodulatory, neuroprotective, organ protective effects, and other anti-aging effects (73–76). Several quercetin derivatives were the representatives of flavonoids, which exhibited a wide range of biological activities by regulating multiple targets and signaling pathways, and they are popular candidate compounds in the field of anti-aging and against aging-related diseases (77). Plant tannins are a group of polyphenols with different molecular weights and complexity. Because of their excellent antioxidant properties, they extended beneficial effects on controlling chronic geriatric diseases, such as cancer, diabetes, hyperlipidemia, and hypertension (78). Ellagic acid, gallic acid, and their derivatives were the main tannins detected in RSGB oral liquid and serum of RSGB-treated mice. Ellagic acid is a concentrated dimer of gallic acid in structure; ellagic acid and gallic acid were usually detected together in extracts (79). They are both potential drugs on the prevention of various oxidative stress-associated diseases due to their strong antioxidative effect (80, 81). Moreover, their methylated, acetylated, and glycosylated derivatives have a more excellent physiological activity (81–83). Many lines of evidence suggest that polyphenols have a facilitative link to probiotics and bile acid metabolism (84). RSGB also contained choline and many nucleotides, such as cytidine, adenine, adenosine, guanine, guanosine, and uridine; all of them were involved in critical physiological functions in organisms. Some were reported to have super anti-aging properties (85–88). RSGB also had higher levels of amino acids and organic acids; they were all nutrients for the body. Above all, the ingredients in RSGB oral liquid and medicated serum had a variety of pharmacological activities, such as antioxidant, immunomodulatory, increasing bile acids metabolism, promoting probiotics, nutrient replenishment, and anti-aging, which corresponded to the pharmacodynamic test results in this study. They are the material basis for the anti-aging effects of RSGB on thyrotoxicosis mice.

**Figure 7:**
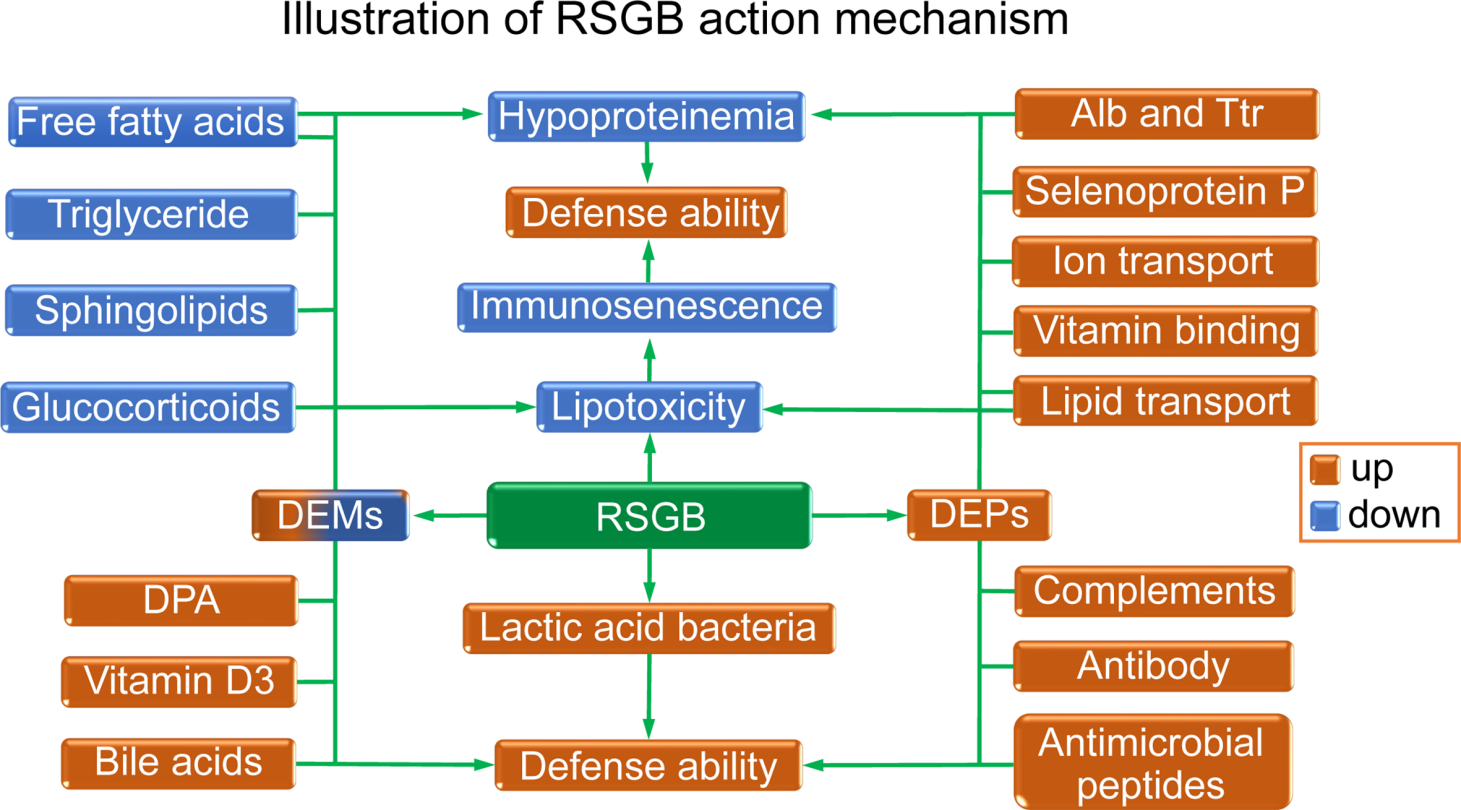
Illustration of the effects and mechanism of Renshen Guben (RSGB) on thyrotoxicosis mice. The RSGB treatment significantly improved hypoproteinemia by upregulating Alb, Ttr, and many functional proteins. The recovery of the above-mentioned proteins not only indicated the improvement of malnutrition but also was beneficial for maintaining immunity and defense ability. The RSGB treatment improved immunosenescence by reducing lipotoxicity. RSGB alleviated lipotoxity by downregulating the level of the lipotoxic lipid-like molecules triglyceride, sphingolipids, glucocorticoids, and some free acids. The RSGB itself also has a direct anti-lipotoxicity effect by its large number of antioxidant components. The upregulation of DPA, vitamin D3, and bile acids was beneficial for maintaining defense capacity, as was the upregulation of lactic acid bacteria in the intestine.

The illustration of the RSGB action mechanism is displayed in **Figure 7**. The RSGB treatment significantly improved hypoproteinemia in thyrotoxicosis mice by the upregulation of Alb, Ttr, and functional proteins. The recovery of the above-mentioned proteins not only indicated the improvement of malnutrition but also was beneficial for maintaining defense ability. The RSGB treatment improved immunosenescence by reducing lipotoxity. RSGB alleviated lipotoxity by decreasing the level of lipotoxic lipids and lipid-like molecules such as free fatty acids, triglyceride, sphingolipids, and glucocorticoids. Moreover, the numerous active ingredients with excellent antioxidant activity in RSGB have direct anti-lipotoxicity effects. The increasing level of protective lipid-like molecules DPA, vitamin D3, and bile acids contributed to the improvement of defense ability. RSGB also upregulated the lactic acid bacteria, which was beneficial for maintaining intestinal defense ability.

Totally, RSGB improved the aging features of thyrotoxicosis mice from the following aspects: increasing the number and function of immune cells, raising the level of immune molecules, reducing lipotoxicity, improving the nutritional status, and regulating the intestinal flora. It suggested that RSGB could restore the resilience of elderly people, protecting them from the risk of infection, improving their prognosis, and reducing their hospital stay, which is of great significance for personal health and social burden. The safety study for 30 consecutive days showed that no toxicity was found. This study verified the anti-aging effect of RSGB on thyrotoxicosis mice and clarified its mechanisms and material basis. In addition to the anti-aging effect obtained by testing the blood indicators, RSGB might also have the organ-protective effect because of its multiple protective effects, and the next studies will examine organ-protective effects to more fully demonstrate the anti-aging effects of RSGB. For a long time, the multi-element and multi-effect systematic treatment strategies of TCM have played an important role in ensuring the health of the Chinese people. It is believed that with the continuous research and understanding of TCM, it will also play a greater role in ensuring global human health in the future.

## Data availability statement

The data presented in the study are deposited in the iProX repository, https://www.iprox.cn/page/PSV023.html;?url=16596818978029yrz, under Password: Vt0R. The 16s rDNA sequencing data are deposited in the GSA repository, https://bigd.big.ac.cn/gsa/browse/CRA007731.

## Ethics statement

The animal study was reviewed and approved by the Animal Care and Use Committee of Shandong Province, China.

## Author contributions

GZ conceived the project. QF designed the experiments. GL, WX, GD, JZ, and YX conducted the experiments. QF and DL wrote the paper. All authors contributed to the article and approved the submitted version.

## Acknowledgments

We thank the Shanghai LuMing Biological Technology Co., Ltd. (Shanghai, China) for providing proteomics and metabonomics services.

## Conflict of interest

Authors QF, GL, WK, GD, JZ, YX, and GZ are employed by Lunan Pharmaceutical Group Co., Ltd.

The remaining author declares that the research was conducted in the absence of any commercial or financial relationships that could be construed as a potential conflict of interest.

## Publisher’s note

All claims expressed in this article are solely those of the authors and do not necessarily represent those of their affiliated organizations, or those of the publisher, the editors and the reviewers. Any product that may be evaluated in this article, or claim that may be made by its manufacturer, is not guaranteed or endorsed by the publisher.
